# A tissue-specific role of membrane-initiated ERα signaling for the effects of SERMs

**DOI:** 10.1530/JOE-21-0398

**Published:** 2022-03-07

**Authors:** Karin L Gustafsson, Sofia Movérare-Skrtic, Helen H Farman, Cecilia Engdahl, Petra Henning, Karin H Nilsson, Julia M Scheffler, Edina Sehic, Ulrika Islander, Ellis Levin, Claes Ohlsson, Marie K Lagerquist

**Affiliations:** 1 Sahlgrenska Osteoporosis Centre, Centre for Bone and Arthritis Research at Institute of Medicine, Sahlgrenska Academy at University of Gothenburg, Gothenburg, Sweden; 2 Department of Rheumatology and Inflammation Research, Institute of Medicine, Sahlgrenska Academy at University of Gothenburg, Gothenburg, Sweden; 3 Division of Endocrinology, Department of Medicine, University of California, Irvine, Irvine, California, USA; 4 Department of Veterans Affairs Medical Center, Long Beach, Long Beach, California, USA; 5 Department of Drug Treatment, Sahlgrenska University Hospital, Region Västra Götaland, Gothenburg, Sweden

**Keywords:** estrogen receptor alpha, selective estrogen receptor modulators, estrogen, palmitoylation, bone, uterus

## Abstract

Selective estrogen receptor modulators (SERMs) act as estrogen receptor (ER) agonists or antagonists in a tissue-specific manner. ERs exert effects via nuclear actions but can also utilize membrane-initiated signaling pathways. To determine if membrane-initiated ERα (mERα) signaling affects SERM action in a tissue-specific manner, C451A mice, lacking mERα signaling due to a mutation at palmitoylation site C451, were treated with Lasofoxifene (Las), Bazedoxifene (Bza), or estradiol (E2), and various tissues were evaluated. Las and Bza treatment increased uterine weight to a similar extent in C451A and control mice, demonstrating mERα-independent uterine SERM effects, while the E2 effect on the uterus was predominantly mERα-dependent. Las and Bza treatment increased both trabecular and cortical bone mass in controls to a similar degree as E2, while both SERM and E2 treatment effects were absent in C451A mice. This demonstrates that SERM effects, similar to E2 effects, in the skeleton are mERα-dependent. Both Las and E2 treatment decreased thymus weight in controls, while neither treatment affected the thymus in C451A mice, demonstrating mERα-dependent SERM and E2 effects in this tissue. Interestingly, both SERM and E2 treatments decreased the total body fat percent in C451A mice, demonstrating the ability of these treatments to affect fat tissue in the absence of functional mERα signaling. In conclusion, mERα signaling can modulate SERM responses in a tissue-specific manner. This novel knowledge increases the understanding of the mechanisms behind SERM effects and may thereby facilitate the development of new improved SERMs.

## Introduction

Estrogen treatment protects against osteoporosis-related fractures, has favorable effects on several metabolic parameters, and alleviates postmenopausal symptoms. However, due to adverse effects, estrogen treatment is avoided. Selective estrogen receptor modulators (SERMs) act as estrogen receptor (ER) agonists in some tissues, and as antagonists in others, have been developed to avoid these adverse effects. SERMs are mainly used to prevent and treat osteoporosis and breast cancer and to alleviate postmenopausal symptoms, but also to maintain a beneficial lipid profile in postmenopausal women (Kung*et al.* 2009, Lewiecki 2009, Yavropoulou*et al.* 2019).

Lasofoxifene (Las) and Bazedoxifene (Bza), which are third-generation SERMs, have agonistic effects in bone and can prevent both vertebral and non-vertebral fractures in humans (Cummings*et al.* 2010, Silverman*et al.* 2012). Animal studies have also shown positive effects of these SERMs at both vertebral and non-vertebral (i.e. long bones) bone sites (Bernardi*et al.* 2014, Borjesson*et al.* 2016). Estrogen treatment increases uterine growth, resulting in an increased risk of cancer in this reproductive organ, and this effect is shown to be dependent on the expression of insulin-like growth factor-1 (*Igf1*) (Adesanya*et al.* 1999, Kashima*et al.* 2009, Hewitt*et al.* 2019). The adverse estrogenic effect on uterine growth can be inhibited by treatment with the SERMs Las and Bza (Crabtree*et al.* 2008), demonstrating antagonistic effects of these SERMs in the uterus in the presence of estrogen, and Las and Bza treatment in postmenopausal women does not result in adverse uterine effects (Ronkin*et al.* 2005, Cummings *et al.* 2010, De Villiers*et al.* 2011). Even though Las and Bza have fewer adverse effects than estradiol, increased risk for venous thrombosis has been reported (Lewiecki 2009). There are today no SERMs available without adverse effects, and more knowledge regarding the mechanisms behind the effects of SERMs in various tissues is therefore needed to be able to develop new improved SERMs.

SERMs exert effects by binding to ERs. ERs can, in addition to nuclear actions, also exert membrane-initiated effects. ERα is the major mediator of many estrogenic effects in the body and palmitoylation of cysteine 451 (C451) in the murine ERα is required for association of the receptor to the membrane (Acconcia*et al.* 2005, Adlanmerini*et al.* 2014, Pedram*et al.* 2014). Mice with a mutated ERα C451 site (C451A mice) are devoid of membrane-associated ERα and can thereby be used as a tool to determine the importance of membrane-initiated ERα (mERα) signaling (Adlanmerini *et al.* 2014, Pedram *et al.* 2014).

Some of the tissue-specificity of SERMs may be attributed to the binding of the SERM-ER complex to tissue-specific co-regulators (Kressler*et al.* 2002). However, since we and others have shown that mERα signaling results in tissue-specific estrogenic effects (Adlanmerini *et al.* 2014, Gustafsson*et al.* 2016, Farman*et al.* 2018, Guivarc’h*et al.* 2018), mERα signaling might also contribute to the tissue-specificity of SERMs. The aim of this study was therefore to determine if mERα signaling affects the action of the SERMs Las and Bza in a tissue-dependent manner in order to improve the understanding of the mechanisms behind the tissue-specificity of SERMs.

## Materials and methods

### Animals

All animal experiments were approved by the Gothenburg Ethical Committee for Animal Research. Transgenic C451A mice with a point mutation at the palmitoylation site C451 in ERα has been described before (Gustafsson *et al.* 2016). The primers used for genotyping of C451A mice were 5ʼ-CTAAACAAGCTTCAGTGGCTCCTAG-3ʼ and 5ʼ-ACCTGCAGGGAGAAGAGTTTGTGGC-3ʼ. The transgenic mice and littermate controls were housed in a standard animal facility under controlled temperature (22°C) and photoperiod (12 h light:12 h darkness cycle) and fed phytoestrogen free pellet diet *ad libitum*(Harlan Rodent Diet, 2016).

### Treatment

Ovariectomy was performed on 12-week-old C451A and WT (control) littermate mice. After one-week recovery the mice received daily subcutaneous injections for 3 weeks with either vehicle (veh; Miglyol 812; OmyaPeralta GmbH), 17β-estradiol-3-benzoate (estradiol (E2); 0.3 μg/mouse/day; Sigma-Aldrich), Lasofoxifene (Las; 8 μg/mouse/day; Sigma-Aldrich), or Bazedoxifene (Bza; 24 μg/mouse/day; Sigma-Aldrich). The doses were chosen based on the substances’ ability to protect against ovariectomy-induced bone loss (Andersson*et al.* 2016, Borjesson *et al.* 2016) and body surface area calculations ensured that the doses of Las and Bza were similar to those used in humans (Reagan-Shaw*et al.* 2008). Surgery was performed under anesthesia with isoflurane (Baxter Medical AB, Kista, Sweden) and Rimadyl (Orion Pharma AB, Animal Health, Sollentuna, Sweden) was given as an analgesic. At the termination, the mice were anesthetized with Ketador/Dexdomitor (Richter Pharma/Orion Pharma), bled, and euthanized by cervical dislocation. Uterus and thymus were collected and weighed. The femur and vertebra L5 were dissected, fixated in 4% paraformaldehyde for 2 days, and stored in 70% ethanol for further analysis.

### Assessment of bone parameters

#### Dual-energy X-ray absorptiometry

Analyses of total body areal bone mineral density (aBMD) were performed using a Lunar PIXImus mouse densitometer (Wipro GE Healthcare).

#### High-resolution microcomputed tomography

High-resolution microcomputed tomography (μCT) analysis was performed on the vertebra (L5) and femur using an 1172 model μCT (Bruker MicroCT, Aartselaar, Belgium) as previously described (Moverare-Skrtic*et al.* 2014). Briefly, in the vertebra, the trabecular and cortical bone in the vertebral body caudal of the pedicles were selected for analysis within a conforming volume of interest (cortical bone excluded for trabecular bone and trabecular bone excluded for cortical bone) commencing at a distance of 4.5 µm caudal of the lower end of the pedicles and extending a further longitudinal distance of 225 µm in the caudal direction. In the femur, the trabecular bone proximal to the distal growth plate was selected for analyses within a conforming volume of interest (cortical bone excluded), commencing at a distance of 650 μm from the growth plate and extending a further longitudinal distance of 134 μm in the proximal direction. The cortical measurements in the femur were performed in the diaphyseal region starting at a distance of 5.2 mm from the growth plate and extending a further longitudinal distance of 134 µm in the proximal direction.

### Real-time PCR

RNA was isolated from uterus using the RNeasy Mini Kit (Qiagen). The RNA was reversed transcribed into cDNA using the High-Capacity cDNA Reverse Transcription kit (Applied Biosystems, Thermo Fisher Scientific). Amplifications were performed using the Applied Biosystem StepOnePlus Real-Time PCR System (ThermoFisher Scientific) and Assay-on-Demand primer and probe sets (ThermoFisher Scientific), labeled with the reporter fluorescent dye FAM. Predesigned primers and probes labeled with the reporter fluorescent dye VIC, specific for 18S ribosomal RNA, were included in the reaction as an internal standard. The assay identification numbers were insulin-like growth factor-1 (*Igf1*: Mm00439559_m1), progesterone receptor (*Pgr*: Mm00435628_m1), lactotransferrin (*Ltf*: Mm00434787_m1), cytokeratin 8 (*Krt8*: Mm04209403_g1), and 18S: (4310893E). The relative gene expression values were calculated using the ΔΔCt method.

### Statistical analyses

In the figures, all individual values are presented with mean (horizontal line) and s.e.m. (vertical lines). In the tables, values are given as mean ± s.e.m. The statistical differences between veh and E2, Las, and Bza were calculated using one-way ANOVA followed by Dunnett’s* post hoc* test separately for each genotype (GraphPad Prism version 9.2.0). To determine if there was a statistically significant difference in the treatment responses between C451A and control mice, the interaction *P* value from two-way ANOVA for each treatment was used. Logarithmic transformations were used when appropriate to ensure normal distribution of data.

## Results

### Las and Bza affect uterine weight in a mERα-independent manner

**Figure 1:**
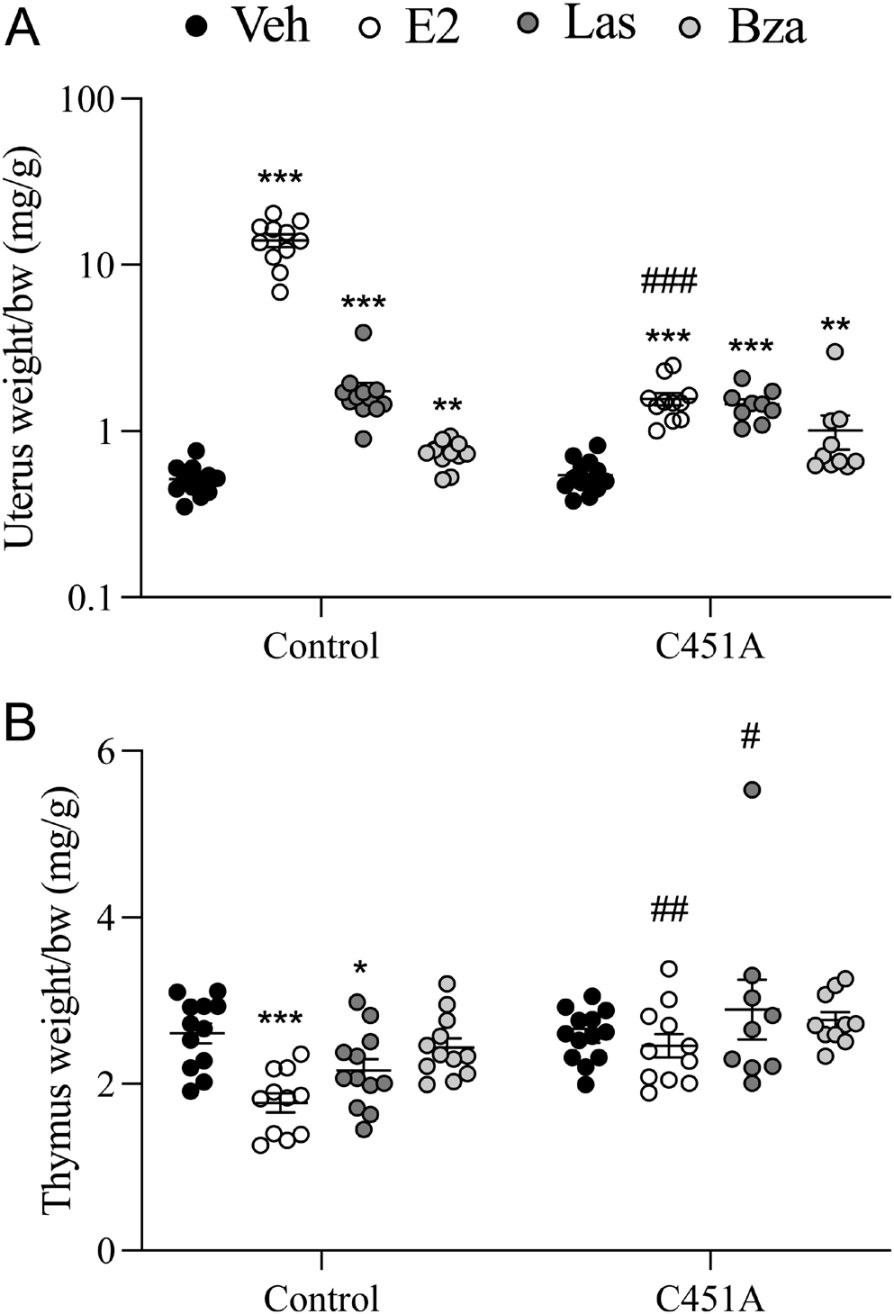
SERM effects on uterus weight are mERα-independent. Twelve-week-old C451A and control female mice were ovariectomized and treated with 17β-estradiol (E2, 0.3 μg/mouse/day), Lasofoxifene (Las, 8 μg/mouse/day), Bazedoxifene (Bza, 24 μg/mouse/day), or vehicle (veh) by subcutaneous injections daily for 3 weeks. Uterus weight/body weight (bw) (A), and thymus weight/bw (B). All individual values are presented with mean (horizontal line) and s.e.m (vertical lines). (*n*= 9–13). ***P  < 0.001, **P  < 0.01, *P  < 0.05, one-way ANOVA, followed by Dunnett’s posthoc test, vs control veh or C451A veh, respectively. ^###^*P*  < 0.001, ^##^*P*  < 0.01, ^#^*P*  < 0.05, Interaction *P* value from two-way ANOVA, for comparison of treatment responses between C451A and control mice.

E2 treatment increased the uterus weight in control mice, as expected, and a small increase in uterus weight was also found in C451A mice (Fig. 1A). However, the E2 effect in C451A mice was significantly decreased compared to the effect in control mice (−93%, *P*  < 0.001, interaction *P*value from two-way ANOVA). In contrast, treatment with the SERMs Las and Bza increased the uterus weight to a similar extent in controls and C451A mice (Fig. 1A). The mRNA expression of *Igf1*, *Pgr*, and *Ltf,* three genes previously shown to be regulated by E2 in uterine tissue (Liu & Teng 1992, Kraus*et al.* 1994, Hewitt*et al.* 2003), were increased after E2 treatment in control mice, as expected (Table 1). E2 treatment increased *Ltf* and *Pgr* expression in the uterus from C451A mice, while *Igf-1* expression was unaffected by E2 in C451A mice (Table 1). The E2 effect on *Pgr* expression was similar between controls and C451A mice, while the effect on *Ltf* expression was significantly decreased in C451A mice compared to the effect in controls (Table 1). Las treatment increased *Igf1*, *Pgr,* and *Ltf* expression similarly in controls and C451A mice, while Bza treatment resulted in increased expression of *Pgr* and *Ltf* in controls and *Igf1* in C451A mice (Table 1). In addition, expression of *Krt8*, an epithelial cell marker (Memarzadeh*et al.* 2010), was significantly increased by E2 in both controls and C451A mice, however, the E2 effect in C451A mice was significantly decreased compared to the effect in control mice (−87%, *P*  < 0.001, interaction *P* value from two-way ANOVA, Table 1). Las treatment increased *Krt8* expression in controls and C451A mice to a similar extent, while Bza had no effect on *Krt8* expression (Table 1).

**Table 1:** mRNA expression in the uterus. Twelve-week-old C451A and control female mice were ovariectomized and treated with 17β-estradiol (E2, 0.3 µg/mouse/day), Lasofoxifene (Las, 8 µg/mouse/day), Bazedoxifene (Bza, 24 µg/mouse/day), or vehicle (veh) by subcutaneous injections daily for 3 weeks.

	Control				C451A			
	Veh	E2	Las	Bza	Veh	E2	Las	Bza
*Igf-1*	2.7 ± 0.2	4.4 ± 0.5^b^	7.7 ± 0.9^a^	2.9 ± 0.2	2.4 ± 0.3	3.3 ± 0.4	6.2 ± 0.9^a^	4.0 ± 0.5^c^
*Pgr*	2.5 ± 0.2	4.5 ± 0.5^a^	5.2 ± 0.3^a^	3.4 ± 0.2^c^	2.8 ± 0.3	5.2 ± 0.5^a^	4.5 ± 0.3^b^	3.2 ± 0.4
*Ltf*	2.3 ± 0.2	372.1 ± 62.5^a^	31.4 ± 3.4^a^	4.3 ± 0.6^b^	3.8 ± 0.7	13.3 ± 2.6^a,d^	38.4 ± 4.7^a^	8.5 ± 3.1
*Krt8*	2.2 ± 0.3	43.4 ± 9.0^a^	12.0 ± 0.9^a^	2.9 ± 0.3	2.8 ± 0.2	9.4 ± 1.3^a,d^	14.6 ± 1.9^a^	3.8 ± 0.9

^a^*P* <0.001, ^b^*P* <0.01, ^c^*P* <0.05, one-way ANOVA, followed by Dunnett’s posthoc test, vs control veh or C451A veh, respectively. ^d^*P* <0.001, interaction *P* value from two-way ANOVA, for comparison of treatment responses between C451A and control mice.

Values (arbitrary unit) are given as mean ± S.E.M. (n=9-13).

### Las affects thymus weight in a mERα-dependent manner

Thymus weight was decreased by E2 treatment in control mice, while no effect was detected in C451A mice (Fig. 1B). The same pattern was seen for Las treatment with a decreased thymus weight in control mice, and no significant effect in C451A mice, while Bza did not affect thymus weight in either controls or C451A mice (Fig. 1B).

### Las and Bza can affect total body fat percent in mice with inactivated mERα signaling

Body weight and lean mass, as measured by dual-energy X-ray absorptiometry (DXA), were unchanged in both control and C451A mice after treatment with E2 or SERMs (Table 2). The percent fat, measured by DXA, was decreased after E2 treatment in control mice, and a decrease was seen also in C451A mice (Table 2). Las or Bza treatments did not affect fat percent in control mice, but both SERM treatments resulted in decreased percent fat in C451A mice (Table 2).

**Table 2:** Body composition of C451A and control mice. Twelve-week-old C451A and control female mice were ovariectomized and treated with 17β-estradiol (E2, 0.3 μg/mouse/day), Lasofoxifene (Las, 8 μg/mouse/day), Bazedoxifene (Bza, 24 μg/mouse/day), or vehicle (veh) by subcutaneous injections daily for 3 weeks. Lean mass and fat percent were measured by DXA. Values are given as mean ± s.e.m. (*n*= 9–13).

	Control				C451A			
	Veh	E2	Las	Bza	Veh	E2	Las	Bza
Body weight (g)	19.9 ± 0.5	19.6 ± 0.4	19.2 ± 0.4	19.2 ± 0.4	20.0 ± 0.7	20.1 ± 0.5	18.9 ± 0.4	19.5 ± 0.4
Lean mass (%)	14.0 ± 0.3	14.5 ± 0.3	13.5 ± 0.2	13.8 ± 0.3	13.9 ± 0.3	14.4 ± 0.4	13.7 ± 0.3	13.9 ± 0.3
Fat (%)	19.2 ± 0.9	14.8 ± 0.5^a^	18.7 ± 0.8	16.8 ± 0.4	20.6 ± 1.4	16.9 ± 0.6^b^	16.3 ± 0.3^b^	17.1 ± 0.6^b^

^a^*P*  < 0.001, ^b^*P* < 0.05, one-way ANOVA, followed by Dunnett’s posthoc test, vs control veh or C451A veh, respectively.

### Las and Bza affect both trabecular and cortical bone in a mERα-dependent manner

**Figure 2:**
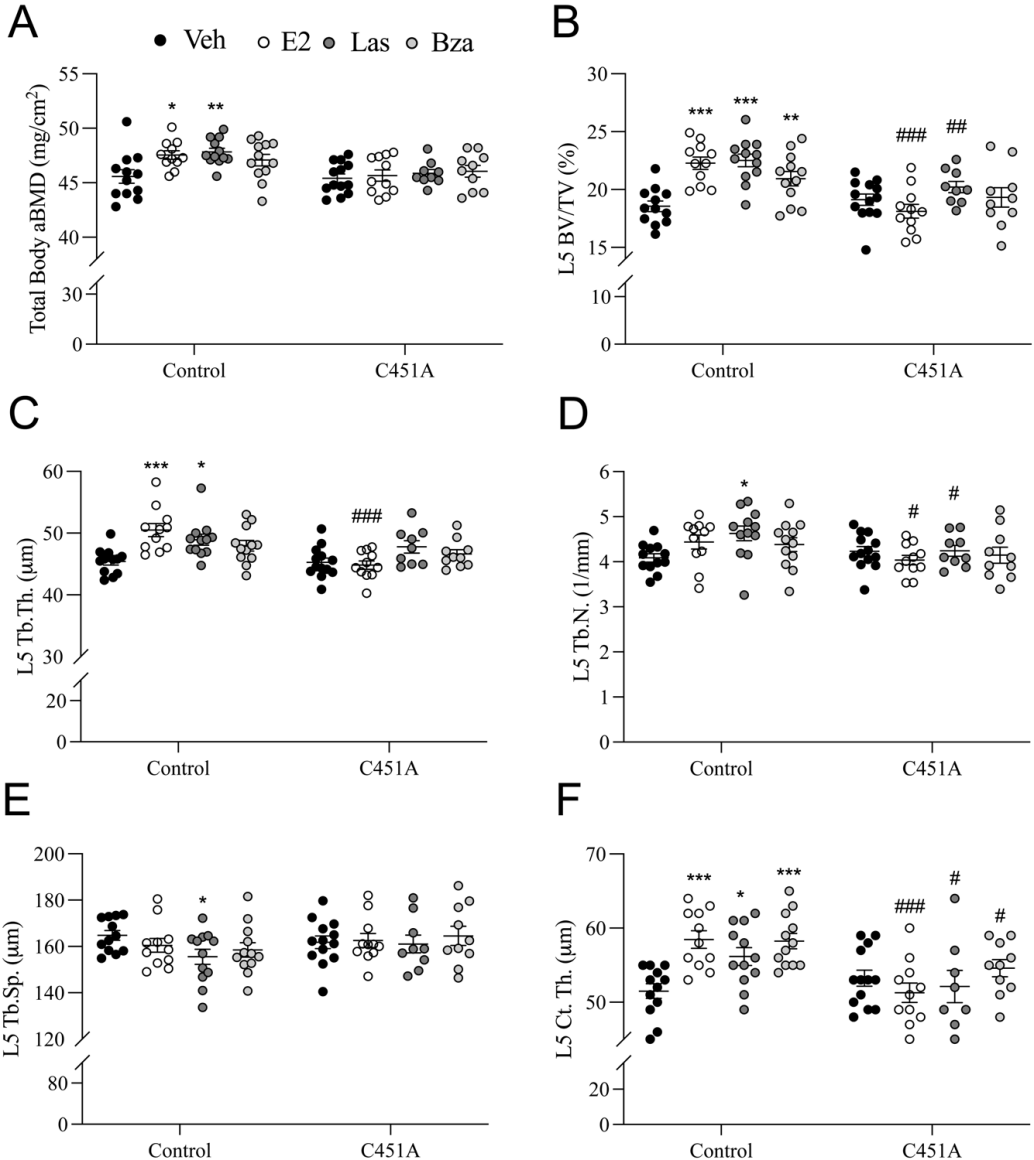
SERM effects in the skeleton are dependent on mERα signaling. Twelve-week-old C451A and control female mice were ovariectomized and treated with 17β-estradiol (E2, 0.3 μg/mouse/day), Lasofoxifene (Las, 8 μg/mouse/day), Bazedoxifene (Bza, 24 μg/mouse/day), or vehicle (veh) by subcutaneous injections daily for 3 weeks. Total body areal bone mineral density (aBMD) (A) measured by DXA. Bone volume per total volume (BV/TV) (B), trabecular thickness (Tb.Th.) (C), trabecular number (Tb.N.) (D), trabecular separation (Tb.Sp.) (E), and cortical thickness (Ct.Th.) (F) in vertebra L5 measured by high-resolution microcomputed tomography. All individual values are presented with mean (horizontal line) and S.E.M. (vertical lines). (*n*= 9–13). ***P  < 0.001, *P  < 0.05, one-way ANOVA, followed by Dunnett’s posthoc test, vs control veh or C451A veh, respectively. ^###^*P*  < 0.001, ^##^*P*  < 0.01,^ #^*P*  < 0.05, Interaction *P* value from two-way ANOVA, for comparison of treatment responses between C451A and control mice.

The skeleton was analyzed by DXA, and both E2 and Las treatments significantly increased total body aBMD in control mice, and there was a tendency to increased total body aBMD also after Bza treatment (*P* = 0.08), while no significant treatment effects were found for any of the treatments in C451A mice (Fig. 2A). Analyses using high-resolution μCT demonstrated that both E2 and SERM treatments increased vertebral trabecular bone volume fraction (BV/TV) in control mice, while no significant effects were seen in C451A mice for any of the treatments (Fig. 2B). Detailed analysis of the trabecular bone in the vertebra showed significantly increased trabecular thickness after E2 treatment in control mice (Fig. 2C), while no significant effects were detected for trabecular number or trabecular separation (Fig. 2D and E). Las treatment increased both trabecular thickness and number and decreased trabecular separation in control mice while Bza treatment had no effect on any of these trabecular parameters in control mice. No significant effects were seen for any of the vertebral trabecular parameters after either E2 or SERM treatments in C451A mice. Cortical bone was also analyzed and both E2 and SERM treatments increased cortical thickness of the vertebrae in control mice, while no significant treatment responses were seen in C451A mice for any of the treatments (Fig. 2F). Femora analyses by μCT showed similar results for both the trabecular and cortical bone as for the vertebrae (Table 3).

**Table 3:** High-resolution microcomputed tomography analysis of the femur . Twelve-week-old C451A and control female mice were ovariectomized and treated with 17β-estradiol (E2, 0.3 µg/mouse/day), Lasofoxifene (Las, 8 µg/mouse/day), Bazedoxifene (Bza, 24 µg/mouse/day), or vehicle (veh) by subcutaneous injections daily for 3 weeks. Values are given as mean ± s.e.m. (*n*= 9–13).

	Control				C451A			
	Veh	E2	Las	Bza	Veh	E2	Las	Bza
BV/TV (%)	12.2 ± 0.8	17.3 ± 0.7^a^	17.3 ± 0.7^a^	13.4 ± 0.8	13.5 ± 0.6	14.7 ± 0.5^d^	16.0 ± 1.0	13.1 ± 0.9
Tb.Th. (μm)	44.1 ± 0.9	49.9 ± 0.6^a^	50.9 ± 0.7^a^	46.9 ± 0.9	45.0 ± 0.8	46.0 ± 0.5	47.4 ± 1.6	46.0 ± 1.2
Tb.N. (1/mm)	2.7 ± 0.1	3.5 ± 0.1^a^	3.4 ± 0.1^b^	2.9 ± 0.1	3.0 ± 0.1	3.2 ± 0.1	3.4 ± 0.1	2.8 ± 0.2
Tb.Sp. (μm)	129.6 ± 0.8	123.5 ± 1.4	125.5 ± 0.9	129.0 ± 0.8	128.4 ± 0.9	126.9 ± 0.9	126.2 ± 1.4	129.7 ± 1.0
Ct.Th. (μm)	193.6 ± 2.6	213.6 ± 2.4^a^	207.2 ± 3.9^b^	205.4 ± 2.5^c^	196.6 ± 2.5	198.1 ± 3.7^d^	204.6 ± 3.5	205.1 ± 2.9

^a^*P* <0.001, ^b^*P* <0.01, ^c^*P* <0.05, one-way ANOVA, followed by Dunnett’s *post hoc* test, vs control veh or C451A veh, respectively. ^d^*P* <0.01, interaction *P* value from two-way ANOVA, for comparison of treatment responses between C451A and control mice.

Ct.Th., cortical thickness; BV/TV, bone volume per total volume; Tb.N., trabecular number; Tb.Sp., trabecular separation; Tb.Th., trabecular thickness.

## Discussion

Estrogen has beneficial effects on several tissues in the body but is not a suitable treatment due to adverse effects. This has prompted the development of SERMs, compounds with agonistic effects in some tissues and antagonistic effects in others. Several SERMs have been approved for clinical use, including Tamoxifen, Lasofoxifene, and Bazedoxifene (Ellis*et al.* 2015). However, clinically used SERMs still have adverse effects, including an increased risk of venous thrombosis and endometrial cancer. Diseases that can be alleviated by SERM treatment, for example postmenopausal osteoporosis, breast cancer, and postmenopausal symptoms, affect a large number of individuals. Therefore, it is of great importance to clarify the mechanisms behind the tissue-specificity of SERM effects in order to aid the development of new SERMs lacking adverse effects. Since we and others have shown that abrogation of mERα signaling results in tissue-specific E2-induced effects, we evaluated if mERα signaling also affects the tissue-specificity of SERMs using C451A mice.

The uterus is a very estrogen-sensitive organ. The proliferative effects of E2 in the uterus can result in endometrial cancer and is one of the unwanted side-effects of E2 treatment. The SERMs Las and Bza both antagonize the E2 effect on uterus weight, although the antagonizing effect of Bza is somewhat greater compared to Las (Crabtree *et al.* 2008). However, in the absence of estrogen, Las has a slight agonistic effect on uterus weight in rodent models, while Bza shows a lack of effect on uterus weight in most (Bernardi *et al.* 2014, Borjesson *et al.* 2016), but not all (Crabtree *et al.* 2008) studies. In the current study, both Las and Bza resulted in a small, but significant increase in uterus weight in control mice. Interestingly, both SERM treatments increased uterus weight to a similar extent in C451A mice as in control mice and uterine *Igf1* mRNA expression was also increased by both SERMs in C451A mice. These data suggest that the effects of Las and Bza on uterine weight involve a mERα-independent increase in *Igf1*mRNA expression.

The importance of mERα signaling for the proliferative effects of E2 treatment in the uterus is not completely clear. In this study, E2 treatment resulted in a small, but significant, increase in uterus weight in C451A mice. However, this E2 effect in C451A mice was significantly attenuated compared to the effect in control mice, demonstrating that a normal E2 response on uterus weight is dependent on mERα signaling, as previously described (Pedram *et al.* 2014, Gustafsson *et al.* 2016). These data show that there is a clear difference in mERα-dependency between E2 and SERMs for the treatment effects on uterus weight.

Nuclear ERα (nERα) signaling is required for normal E2 effects on the uterus. This statement is supported by studies using mice with a deletion of activation function two in ERα, which is required for nuclear actions of ERα (Borjesson*et al.* 2011, Adlanmerini *et al.* 2014), and mice lacking nERα signaling, while still having intact mERα signaling (Pedram*et al.* 2009). Both these mouse models have uteri that are unresponsive to E2 treatment. These data, together with the present and previous (Pedram *et al.* 2014, Gustafsson *et al.* 2016) findings of mERα-dependent E2 effects in the uterus, suggest that both nuclear and membrane-initiated ERα actions are important for optimal estrogenic regulation of the uterus and that there is a cross-talk between these signaling mechanisms in this tissue.

Normal E2 effects on uterine *Igf1* gene expression are known to require nuclear ERα action (Hewitt*et al.* 2010), and nuclear ERα action has also been shown to affect transcription of both *Pgr* and *Ltf* (Liu & Teng 1992, Kraus *et al.* 1994). Interestingly, the present study confirmed the finding by Pedram* et al* that normal E2 treatment effects on *Igf1* and *Ltf* mRNA expression is dependent on mERα signaling (Pedram *et al.* 2014). Thus, both mERα signaling and nERα actions are important for the E2 regulation of uterine expression of *Igf1* and *Ltf*. In contrast, the E2 effect on the uterine expression of *Pgr* was found to be independent of mERα signaling, demonstrating that E2 effects on the uterus involve both mERα-dependent as well as mERα-independent actions.

In contrast to the current study and other reports showing that a normal E2 response on uterus weight is dependent on functional mERα signaling (Pedram *et al.* 2014, Gustafsson *et al.* 2016), there are also studies showing that the E2 response on uterus weight is independent of mERα signaling (Adlanmerini *et al.* 2014, Vinel*et al.* 2016). The discrepancies in mERα dependency for uterine E2 responses between studies may be caused by differences in stage of development, since ovx was performed in young, 4-week-old, females in the studies demonstrating mERα-independent effects (Adlanmerini *et al.* 2014, Vinel *et al.* 2016), while the ovx was performed at about three months of age in the studies demonstrating mERα-dependent effects (Pedram *et al.* 2014, Gustafsson *et al.* 2016). The discrepancy in mERα dependency might also be caused by differences in the two models used. Even though the same mutation has been introduced, differences in the development of the models might affect the E2 responses in the uterus (Adlanmerini *et al.* 2014, Pedram *et al.* 2014).

A limitation of the current study is the lack of histological examination of the uterus to determine the cause of the increased uterine weight after E2 and SERM treatments. To evaluate whether effects on epithelial cells might be involved, we analyzed gene expression of *Krt8*, an epithelial cell marker previously shown to be increased by E2 treatment in the uterus (Helvering*et al.* 2005, Memarzadeh *et al.* 2010). Interestingly, the E2 treatment effect on *Krt8* expression was found to be highly dependent on mERα signaling, similar as seen for the E2 effect on uterine weight. In addition, the Las treatment effect on *Krt8* expression was clearly mERα independent, similar as seen for the Las effect on uterine weight. These data suggest that effects on epithelial cells might be involved in the increased uterine weight seen after E2 and Las treatments, however further studies are needed to fully elucidate the causes of the effects on uterine weight seen after E2 and SERM treatments.

Fractures caused by decreased bone mass are a major health problem and cause suffering for patients and great costs for society. Las and Bza treatments reduce the risk of both non-vertebral and vertebral fractures (Silverman*et al.* 2008, Cummings*et al.* 2010), and it is therefore important to learn more about the mechanisms behind their bone-sparing effects in order to aid the development of new SERMs that reduce the risk of fractures. In this study, we evaluated the importance of mERα signaling for the effects of SERMs on both trabecular and cortical bone. Cortical bone comprises about 80% of the total bone mass and this bone compartment is important for skeletal strength, not only in long bones but also in vertebrae (Roux*et al.* 2021). We found that treatment with Las and Bza increased cortical thickness in the vertebrae of control mice to a similar extent as E2, while no significant effects were detected in the C451A mice for neither E2 nor any of the SERM treatments. The same pattern was seen when we analyzed the cortical bone in the femur, demonstrating that cortical bone in both the axial and the appendicular skeleton is dependent on mERα signaling for a normal response to E2, Las, and Bza. We also analyzed the trabecular bone compartment in both vertebrae and femora and found a similar pattern as for the cortical bone, where the effects seen after E2 or SERM treatments in control mice were absent in the C451A mice. These data demonstrate that mERα signaling is highly involved in the regulation of both the cortical and the trabecular bone compartments in the skeleton by Las and Bza treatments and that this signaling pathway is interesting when considering the development of new SERMs for treatment against bone loss. We and others have previously shown that E2 treatment results in significant effects on both cortical and trabecular bone parameters in C451A mice (Gustafsson *et al.* 2016, Vinel *et al.* 2016, 2018). However, in these studies the E2 effects in C451A mice were significantly decreased compared to the E2 effects in control littermates, supporting the notion that mERα is required for a normal E2 response in the skeleton. In the current study, we did not detect any significant effect of E2 treatment on any of the evaluated bone parameters in the C451A mice, and a possible explanation to this discrepancy may be the difference in administration route used compared to the studies where E2 elicited a significant response on bone parameters in C451A mice (Gustafsson *et al.* 2016, Vinel *et al.* 2016, 2018). In this study, we used daily subcutaneous injections while the other studies used subcutaneous pellets. It has been shown that differences in administration route can affect the circulating E2 levels during the experiment (Ingberg*et al.* 2012), and pellet treatment has been shown to result in higher serum E2 levels compared to a corresponding dose administered via subcutaneous injections during the first 3 weeks of treatment (Ström*et al.* 2008). Thus the importance of mERα signaling might be dose dependent, with higher E2 levels leading to an increase in mERα-independent effects.

In this study, we also evaluated the importance of mERα signaling for the effects of SERMs and E2 treatment on thymus and fat mass, two tissues known to be affected by E2 and SERM treatment (Ke*et al.* 1998, Cooke & Naaz 2004, Stubbins*et al.* 2012, Kim*et al.* 2014, Borjesson *et al.* 2016). E2 and Las, but not Bza, are known to induce thymic atrophy (Borjesson *et al.* 2016), and in the current study, both E2 and Las treatments resulted in thymic atrophy in control mice. E2 treatment was not able to induce thymic atrophy in C451A mice, in line with previous studies (Gustafsson *et al.* 2016), and this lack of response in C451A mice on thymus weight was also seen after treatment with Las. Thus, treatment effects of the SERM Las on thymus is dependent on functional mERα signaling.

E2 treatment suppresses fat development and results in decreased fat content in both humans and rodent models (Gambacciani*et al.* 1997, Cooke & Naaz 2004, Stubbins *et al.* 2012), and the SERMs Las and Bza have been shown to have estrogen agonistic effects on adipose tissue leading to a decrease in body fat (Ke *et al.* 1998, Kim *et al.* 2014). However, in this study, we only saw a significant reduction in total body fat percentage in control mice after E2 treatment. Interestingly, the fat percentage was significantly decreased in C451A mice, not only after E2 treatment, as previously described (Gustafsson *et al.* 2016) but also after both Las and Bza treatments. Thus, Las and Bza treatment can reduce the fat percentage in mice lacking mERα signaling. We have previously shown that the E2 treatment effect on fat percentage is mERα-independent (Gustafsson *et al.* 2016), but this is the first study showing that SERMs can affect fat percentage in the absence of functional mERα signaling. We did not find a significant effect of SERM treatment on fat percentage in control mice, indicating that mERα signaling might mediate an inhibitory effect on fat after SERM treatment. It has been demonstrated that both C451A mice, and mice in which only the mERα signaling is intact, have increased abdominal fat mass, suggesting that both mERα and nERα play a role in fat regulation (Pedram*et al.* 2016). Furthermore, it was recently shown that mERα and nERα collaborate to suppress adipogenesis by inhibition of PPARγ expression which subsequently results in diminished commitment of stem cells to adipogenesis and reduced number of adipocytes (Ahluwalia*et al.* 2020), also supporting a role for mERα signaling in the regulation of fat mass. Additional studies are needed to fully understand the mechanism behind the mERα-independent effects on fat percentage reported in this study.

In summary, SERM effects were found to be mERα-independent in the uterus, which is in sharp contrast to the substantial mERα-dependency seen by E2 treatment in this organ. In contrast, both SERM and E2 effects on the skeleton and thymus were found to be dependent on mERα signaling, while effects on fat percentage were present in mice with inactivated mERα signaling. Thus, mERα signaling can modulate responses to SERMs in a tissue-specific manner. This novel knowledge regarding signaling mechanisms behind SERM effects in various tissues may aid the development of new SERMs with less adverse events.

## Declaration of interest

C O has two patent/patent applications in the field of probiotics and bone health. The other authors have nothing to disclose.

## Funding

This work was supported by the Swedish Research Council (2017-01286), the Swedish state under the agreement between the Swedish government and the county councils (ALF-agreement) (ALFGBG721581), the Gustaf V 80-years fund (FAI-2018-0466), the Swedish Rheumatism Association (R664771), the Emil and Wera Cornell Foundation (100.Islander), the IngaBritt and Arne Lundberg Foundation (LU2017-0076), the Ragnar Söderberg’s Foundation (M133/12), the Knut and Alice Wallenberg Foundation (2015-0317), and the Novo Nordisk http://dx.doi.org/10.13039/501100004191 Foundation (26844).

## Author contribution statement

K L G, C O and M K L conducted the study design. K L G, S M S, H H F, C E, P H, K H N, J M S, E S, U I and M K L were responsible for acquisition of data and K L G, M K L, E R L, and C O performed the analysis and interpretation of data. M K L, K L G and C O wrote the main manuscript text and K L G and M K L prepared the figures. All authors reviewed the manuscript.
